# Proton pump inhibitor‐induced large gastric polyps can regress within 2 months after discontinuation: Experience from two cases

**DOI:** 10.1002/deo2.70090

**Published:** 2025-02-26

**Authors:** Tomoki Inaba, Kenji Yamauchi, Shigenao Ishikawa, Hugh Shunsuke Colvin, Koichi Izumikawa, Kumiko Yamamoto, Sakuma Takahashi, Masaki Wato, Satoko Nakamura, Seiji Kawano

**Affiliations:** ^1^ Department of Gastroenterology Kagawa Prefectural Central Hospital Kagawa Japan; ^2^ Department of Pathology Kagawa Prefectural Central Hospital Kagawa Japan; ^3^ Department of Gastroenterology and Hepatology Okayama University Graduate School of Medicine, Dentistry, and Pharmaceutical Sciences Okayama Japan

**Keywords:** esophagogastroduodenoscopy, fundic gland polyps, gastric acidity, gastroesophageal reflux disease, proton pump inhibitors

## Abstract

The long‐term use of proton pump inhibitors (PPIs) can induce fundic gland polyps (FPs) in the stomach, sometimes leading to numerous large FPs (LFPs). Although PPI discontinuation can reduce LFP size and number, the underlying process remains unstudied.

A 63‐year‐old woman on esomeprazole (20 mg daily for 10 years) was scheduled for endoscopic LFP removal. After PPI discontinuation, her LFPs regressed to <10 mm within 35 days. A 60‐year‐old male physician on rabeprazole (10 mg daily for 12 years) had LFPs detected via esophagogastroduodenoscopy screening. He opted for weekly esophagogastroduodenoscopy with pathological evaluations to monitor changes post‐discontinuation.

One week after PPI withdrawal, gastric juice acidity and viscosity increased, with erosion observed on nearly all LFP surfaces. By day 35, all LFPs regressed and resembled sporadic FPs. This study demonstrated that PPI‐induced LFPs regress within a short period post‐discontinuation and suggests that LFP volume reduction is linked to gastric environment changes, particularly increased acidity.

## INTRODUCTION

Proton pump inhibitors (PPIs) are effective against acid‐related diseases and are relatively safe. However, the long‐term use of PPI has been reported to cause fundic gland polyps (FPs).^1^ Although there are many studies on PPI and the occurrence of FP, there are very few reports that describe the changes in FPs after the PPI discontinuation.^2^, ^3^ In the two cases observed, where the Cytochrome P450 2C19 phenotype was classified as a non‐poor metabolizer, no FPs were present before PPI administration. However, after > 10 years of PPI treatment, multiple large FPs (LFPs) developed. Following PPI discontinuation, the size and number of LEPs decreased. This is the first study to report in detail the changes in PPI‐induced FPs after the discontinuation of PPI therapy.

In this report, we define FPs larger than 10 mm as “LFPs.”

## CASE REPORT

### Case 1

**FIGURE 1:**
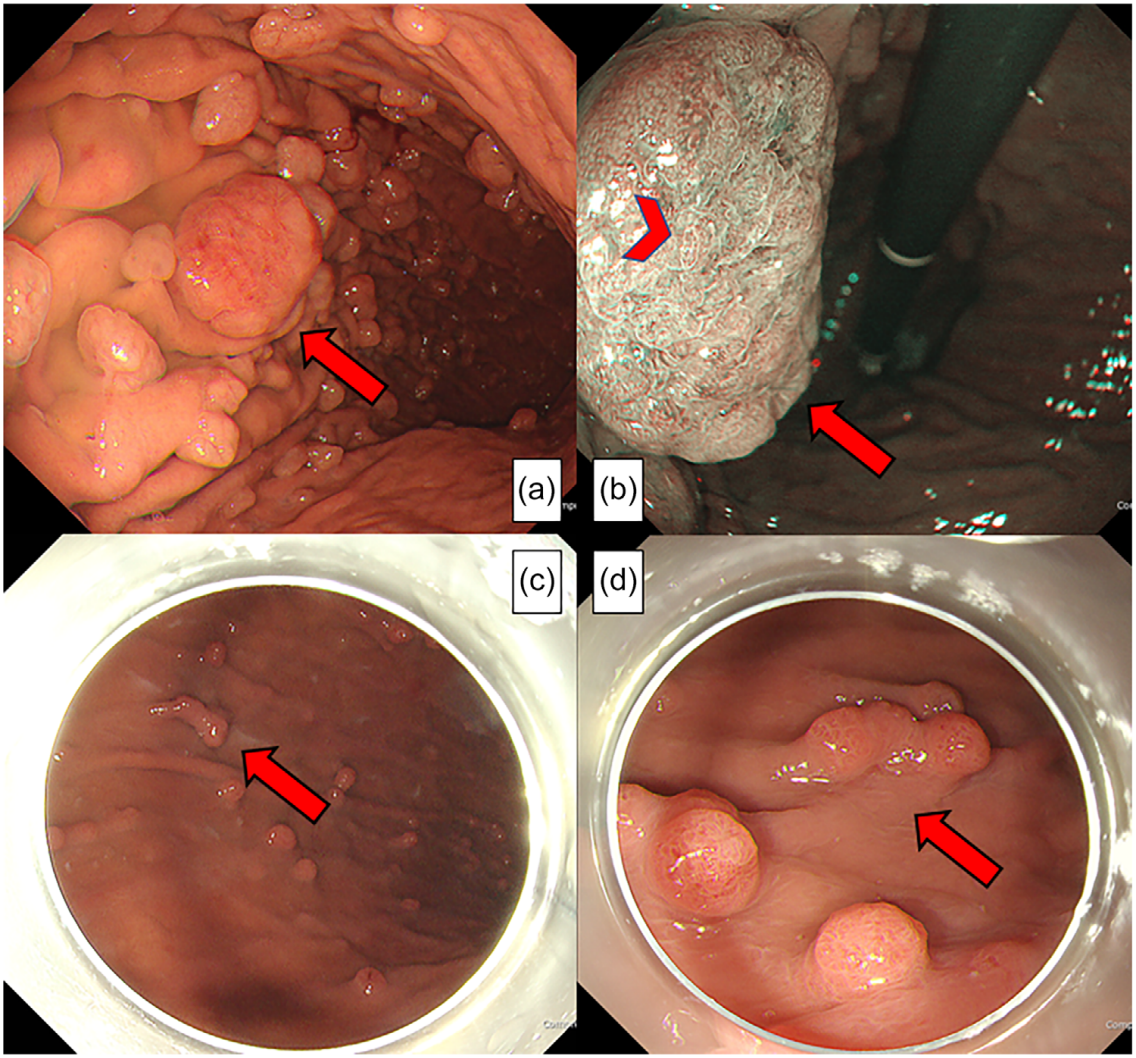
Multiple large polyps, including a 25‐mm polyp (arrow) targeted for endoscopic resection (a). A narrow‐band image revealed an irregular surface with reduced vascularity (arrowhead) in the largest polyp (b). By day 35 after proton pump inhibitor (PPI) discontinuation, the target large fundic gland polyp (arrow) had decreased in size to < 10 mm (c, d).

A 63‐year‐old Japanese woman who had been taking esomeprazole (20 mg daily) for 10 years to treat gastroesophageal reflux disease (GERD) without *Helicobacter pylori* infection underwent esophagogastroduodenoscopy (EGD), which revealed LFPs in the gastric body (Figure 1a). The largest polyp, measuring 25 mm (arrow), had an irregular surface with reduced vascularity, prompting a decision for endoscopic resection (Figure 1b). The patient discontinued esomeprazole the day after the initial endoscopy, and no alternative medication was prescribed for GERD. After 35 days, endoscopic resection was initiated using a transparent hood; however, the procedure was discontinued as the target LFP had shrunk to <10 mm (Figure 1c,d).

### Case 2

A 60‐year‐old male physician who had been taking rabeprazole (10 mg daily) for 12 years to treat GERD without *H. pylori* infection underwent EGD screening, which revealed LFPs in the gastric body. This physician opted to closely monitor the changes in LFPs following PPI discontinuation. Two gastroenterologists determined the observational study protocol as follows:

On the day of the EGD, serum gastrin and pepsinogen levels were measured before the procedure. After discontinuing PPI, the patient underwent weekly EGDs with pharyngeal anesthesia after a 10‐h fast and 3 h of water restriction.

The same endoscopist performed each examination using the same endoscope (GIF‐1200 N; Olympus) and recorded the endoscopic images. A 5 mm yellow circular marker was placed near the two largest FPs, which were designated as targets for follow‐up observation, and images were captured. Two endoscopists subsequently reviewed the recorded footage, counted the number of LFPs in the stomach at each endoscopic examination, and determined the sizes of the two target LFPs.

Gastric juice was collected endoscopically through a polyethylene tube inserted into the suction channel of the endoscope and was used for bacterial culture and pH measurement. The pH of the samples was measured using LAQUAtwin pH‐33B (HORIBA, Ltd.) in another laboratory. During each endoscopic examination, biopsy specimens were obtained from the same LFP, which was not one of the two largest FPs designated as observational targets.

**FIGURE 2:**
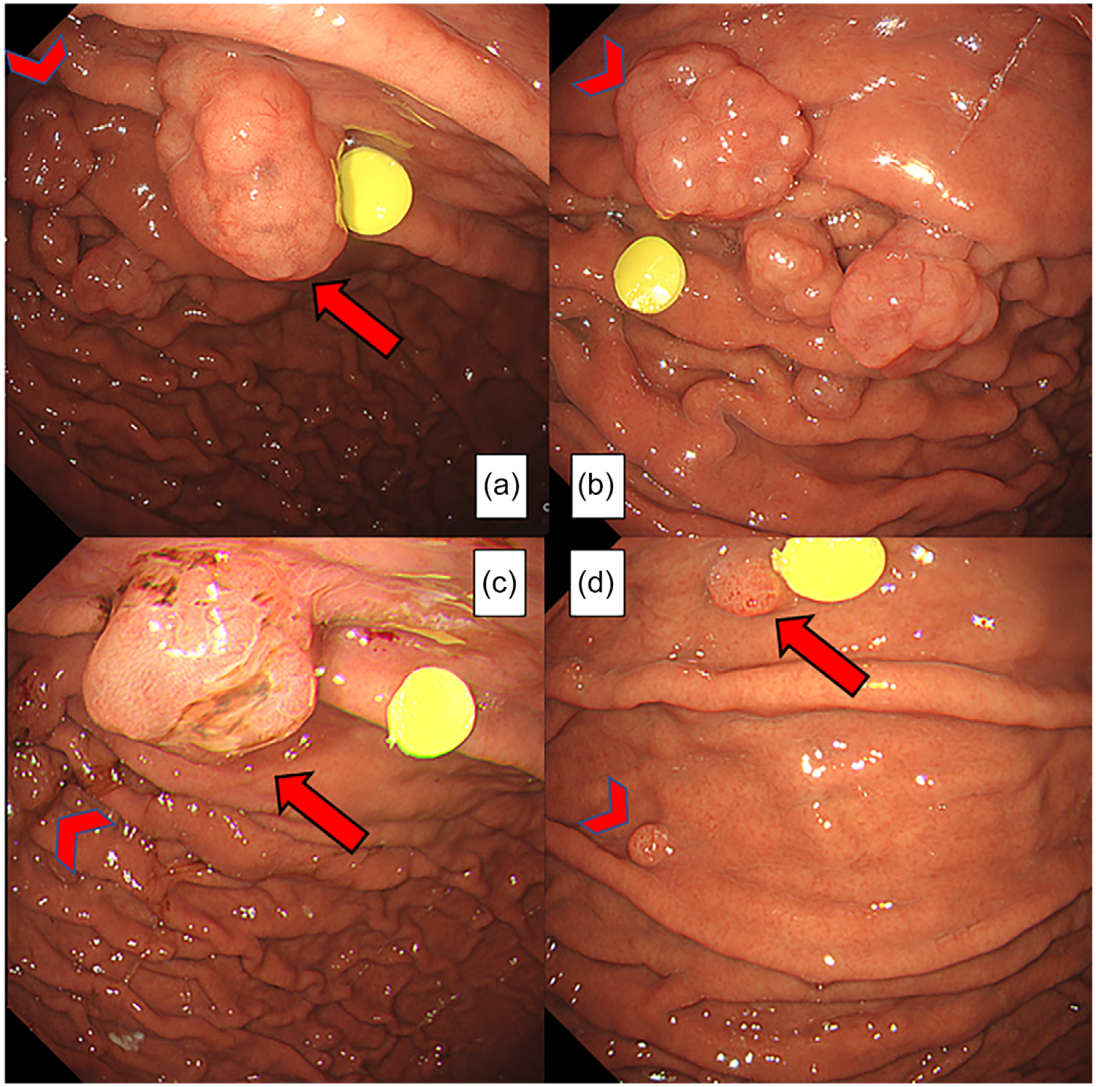
A 20‐mm polyp A (arrow) and a 15‐mm polyp B (arrowhead) are designated as targets for follow‐up observation (a, b). By day 14 after proton pump inhibitor discontinuation, polyp A (arrow) had decreased in size, with surface erosion, and the gastric mucus appeared highly viscous (c). By day 49, both lesions had regressed to <5 mm (d).

The clinical course was as follows: On day ‐1, EGD was performed prior to PPI discontinuation, and a 20‐mm polyp A (arrow) and a 15‐mm polyp B (arrowhead) were chosen for follow‐up observation (Figure 2a,b). A total of 15 LFPs were found in the stomach. On day 0, rabeprazole was discontinued, and no alternative medication was prescribed for GERD. By day 7, polyp A remained the same size, but its bulge had reduced, whereas Polyp B and the surrounding polyps showed surface erosion. The gastric juice appeared viscous (Figure S1). By day 14, both lesions developed erosions on their surfaces, and their sizes decreased. The gastric mucus became extremely viscous (Figure 2c). By day 49, both lesions had regressed to < 5 mm and endoscopic follow‐up was discontinued (Figure 2d).

**FIGURE 3:**
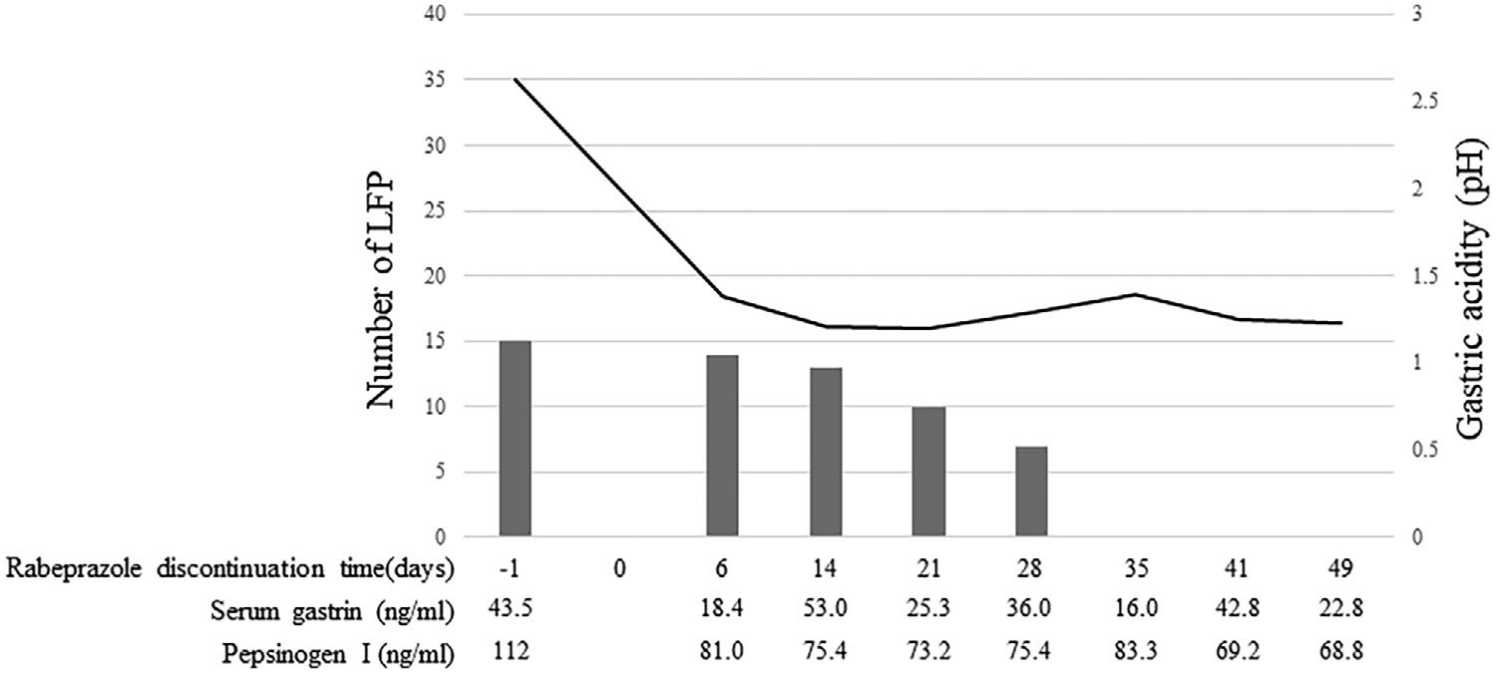
Changes in the pH of gastric juice, the number of large fundic gland polyps (LFPs), and the values of pepsinogen I, pepsinogen II, and gastrin.

Changes in gastric juice pH, the number of LFPs, and serum levels of pepsinogen I, pepsinogen II, and gastrin are shown in Figure 3. Bacterial cultures of the gastric juice collected from each EGD were negative.

**FIGURE 4:**
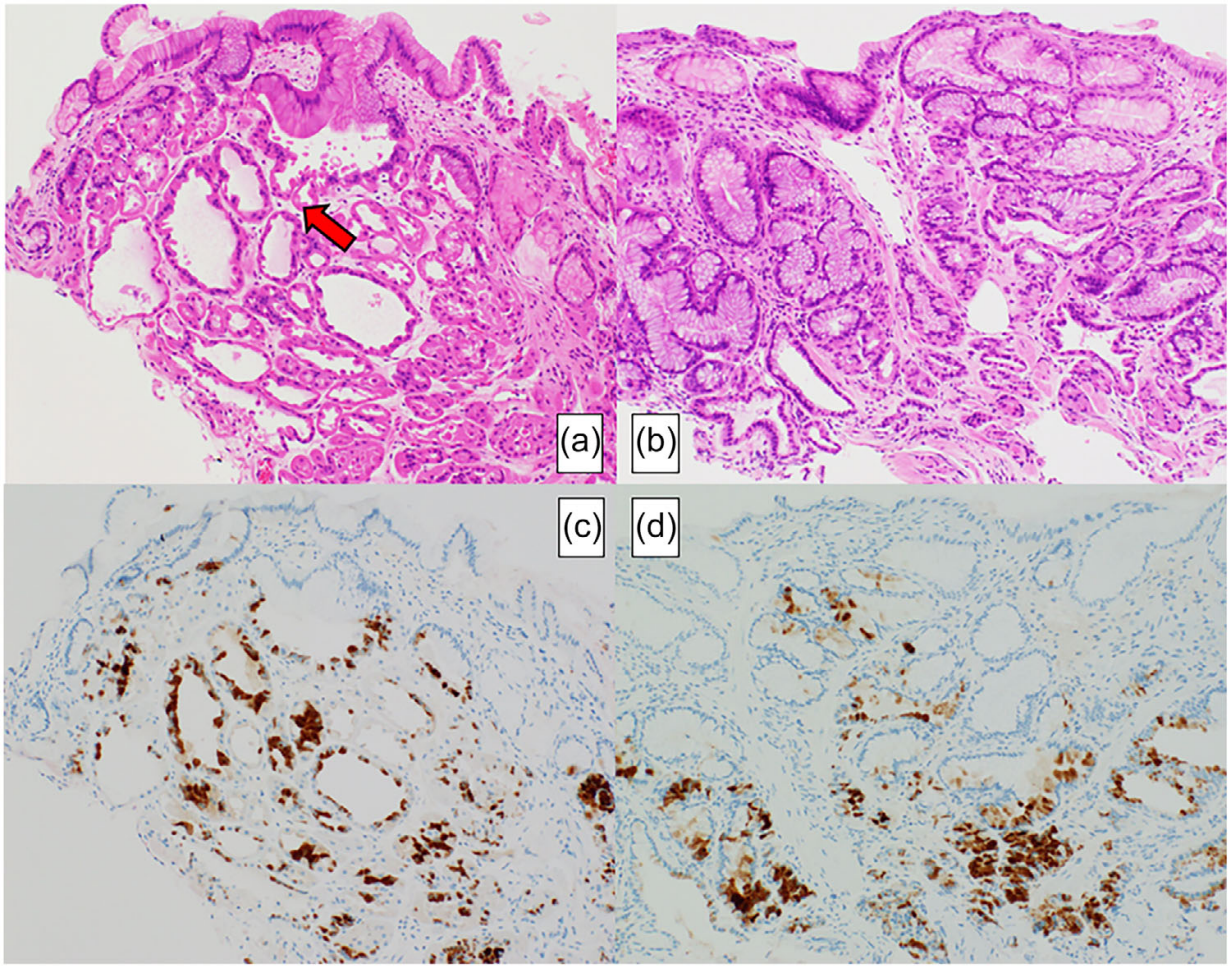
Pathological findings: Cystically dilated fundic glands, lined by attenuated chief cells and parietal cell protrusions (arrow), observed during proton pump inhibitor administration (a) disappeared by day 28 (b). The expression of pepsinogen I in the fundic glands during proton pump inhibitor (PPI) administration (c) decreased by day 28 (d). Magnification (×100).

Pathological findings of LFPs revealed that cystically dilated fundic glands, lined by attenuated chief cells and parietal cell protrusions, which were observed during PPI administration (Figure 4a), disappeared by day 28 after PPI discontinuation (Figure 4b). In addition, pepsinogen I expression, which was present during PPI administration (Figure 4c), decreased by day 28 (Figure 4d).

PAS staining demonstrated that during PPI administration and after its discontinuation (except 3 weeks post‐discontinuation, when it was comparable), the amount of mucus in the fundic gland was higher than that in the non‐polyp site. Following PPI discontinuation, the amount of mucus temporarily decreased in both sites; however, the fluctuation was less pronounced in the non‐polyp site (Figures S2 and S3). The number of mucous cells in the fundic glands decreased in polyp and non‐polyp sites at 3 weeks and 1 week after PPI discontinuation, respectively. Mucous cells increased in polyp sites following their reduction but did not increase in non‐polyp sites.

## DISCUSSION

Although PPI discontinuation can lead to LFP regression, the underlying process remains unclear. Weekly endoscopic evaluations showed that PPI‐induced LFPs decreased in size due to surface erosions and regressed within a short period post‐discontinuation.

PPIs increase serum pepsinogen I and II and gastrin levels.^4^ Theoretically, elevated gastric pH stimulates gastrin secretion, which promotes acid production. In Case 2, serum gastrin levels were not elevated during PPI use, consistent with reports that gastrin does not drive PPI‐induced FPs.^5^

The mechanism of pepsinogen I elevation due to PPIs remains unclear. Omeprazole suppresses pepsinogen production genetically but also inhibits secretion, causing pepsinogen accumulation in the mucosa.^6^ Pathological examination of Case 2′s LFP biopsy showed increased pepsinogen I expression during PPI therapy.

The fasting gastric juice pH of *H. pylori*‐negative volunteers is 1.9 (1.3–5.8), rising to 4.3 (1.3–6.8) after 2 weeks of omeprazole (20 mg).^7^ In Case 2, despite a single‐point evaluation, gastric pH dropped from 2.62 to 1.38 post‐discontinuation. Reports suggest gastric pit‐parietal cells exfoliate into the lumen due to strong acid stimulation, with cytoplasmic processes sealing by the adjacent surface mucous cells.^8^ This could lead to mucosal injury, but Case 2′s biopsy 1‐week post‐discontinuation showed no pathological exfoliation.

PPIs reduce gastric mucus viscosity via pH‐dependent mechanisms.^9^ In Case 2, mucus on LFPs became highly viscous after discontinuation, suggesting gastric instability. PAS staining showed mucus volume in LFP fundic glands, which increased during PPI therapy, decreased 3 weeks post‐discontinuation, and then rose again. Since soluble mucus does not accurately reflect adherent mucus synthesis, the relationship between mucus viscosity and fundic gland secretion remains unclear. However, PPI discontinuation altered the LFP surface.

Surface vessel assessment was not performed in all LFPs, but in Case 1, a reduction in LFP blood vessels was confirmed. Along with glandular dilation and parietal cell hyperplasia, reduced blood flow likely increased fragility. Post‐discontinuation changes, such as elevated gastric acid secretion and pepsinogen I release, may disrupt LFP homeostasis before the gastric mucosa adapts to increased acidity, possibly leading to LFP autolysis by gastric juice.

Because chief and parietal cell turnover is slow, the healthy fundic gland observed in LFP biopsy tissue 28 days post‐discontinuation likely represents a stabilized polyp portion, following regression of vulnerable, cystically dilated glands, rather than regeneration.

This study suggests LFP regression is linked to gastric environmental changes, particularly increased acidity, but further research is needed to clarify PPI‐induced LFP regression mechanisms, especially regarding FP pathophysiology.

Previous reports^2^, ^3^ confirmed LFP regression via EGD at 8 and 10 months post‐PPI discontinuation, with no surface erosions, implying damage resolution. Our early EGD evaluations allowed us to identify the timing of polyp regression.

Since PPI‐related FPs with low‐grade dysplasia do not progress to high‐grade dysplasia or adenocarcinoma,^10^ PPI cessation is not always necessary. However, this study provides valuable insights for patients and clinicians managing LFPs. Most PPI users cannot discontinue therapy due to acid‐related disease symptom control, making large‐scale clinical studies on LFP changes post‐discontinuation difficult. Because Case 2 involved the first author, frequent endoscopic follow‐ups were feasible, overcoming ethical and financial limitations.

In conclusion, we observed two cases of PPI‐induced LFPs regressing within 2 months post‐discontinuation. Serial EGD examinations clearly demonstrated morphological changes in LFPs and gastric environment alterations during the early stages of PPI discontinuation.

## CONFLICT OF INTEREST STATEMENT

None.

## ETHICS STATEMENT

The Ethics Committee of the Kagawa Prefectural Central Hospital approved this study (number: 1224).

## PATIENT CONSENT STATEMENT

Written informed consent was obtained from each patient before the procedure.

## CLINICAL TRIAL REGISTRATION

N/A.

## Supporting information




**Figure S1** On day 7 after PPI discontinuation, polyp A (arrow) and B (arrowhead) remained unchanged in size; however, erosion was observed on the surface of polyp B, and viscous gastric juice was attached to the polyp.

**Figure S2** PAS staining of a biopsy specimen from LFP showed that mucus volume in the fundic glands decreased 3 weeks after PPI discontinuation and subsequently increased. (a) During PPI therapy. (b–d) One, three, and 4 weeks after PPI discontinuation, respectively. Magnification (×100).

**Figure S3** PAS staining of a biopsy specimen from a non‐polyp site showed that mucus volume in the fundic glands decreased 1 week after PPI discontinuation and did not increase thereafter. (a) During PPI therapy. (b–d) One, three, and 4 weeks after PPI discontinuation, respectively. Magnification (×100).
