# Supplementary figures and images for: Proton pump inhibitor‐induced large gastric polyps can regress within 2 months after discontinuation: Experience from two cases
Source: DEN Open. 2025 Feb 26;5(1):e70090. doi: 10.1002/deo2.70090 (PMC11865012; doi:10.1002/deo2.70090)

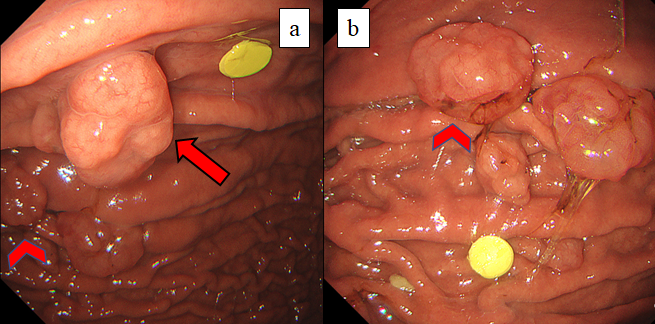

Supplement: Supplementary file 1 — Figure S1 On day 7 after PPI discontinuation, polyp A (arrow) and B (arrowhead) remained unchanged in size; however, erosion was observed on the surface of polyp B, and viscous gastric juice was attached to the polyp. [file DEO2-5-e70090-s003.tif]

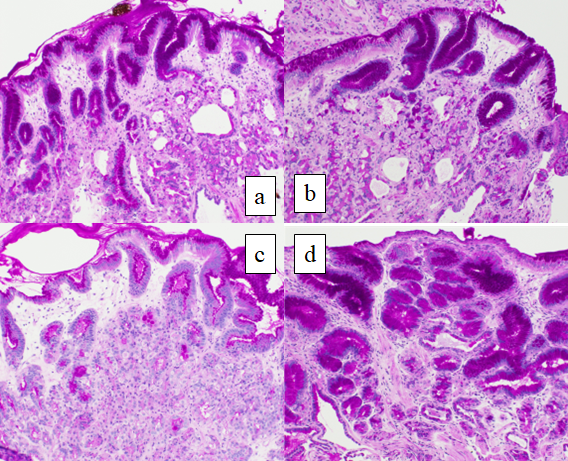

Supplement: Supplementary file 2 — Figure S2 PAS staining of a biopsy specimen from LFP showed that mucus volume in the fundic glands decreased 3 weeks after PPI discontinuation and subsequently increased. (a) During PPI therapy. (b–d) One, three, and 4 weeks after PPI discontinuation, respectively. Magnification (×100). [file DEO2-5-e70090-s002.tif]

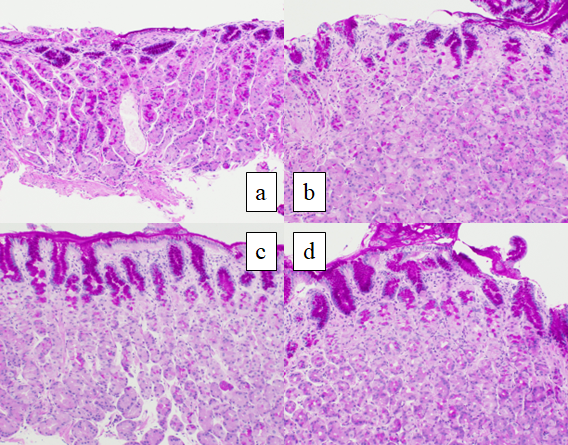

Supplement: Supplementary file 3 — Figure S3 PAS staining of a biopsy specimen from a non‐polyp site showed that mucus volume in the fundic glands decreased 1 week after PPI discontinuation and did not increase thereafter. (a) During PPI therapy. (b–d) One, three, and 4 weeks after PPI discontinuation, respectively. Magnification (×100). [file DEO2-5-e70090-s001.tif]
